# Tumor accomplice: T cell exhaustion induced by chronic inflammation

**DOI:** 10.3389/fimmu.2022.979116

**Published:** 2022-09-02

**Authors:** Liguang Fang, Kunjing Liu, Cun Liu, Xiaomin Wang, Wenzhe Ma, Wenhua Xu, Jibiao Wu, Changgang Sun

**Affiliations:** ^1^ College of First Clinical Medicine, Shandong University of Traditional Chinese Medicine, Jinan, China; ^2^ College of Traditional Chinese Medicine, Weifang Medical University, Weifang, China; ^3^ Department of Inspection, The Medical Faculty of Qingdao University, Qingdao, China; ^4^ State Key Laboratory of Quality Research in Chinese Medicine, Macau University of Science and Technology, Macau, Macao SAR, China; ^5^ College of Traditional Chinese Medicine, Shandong University of Traditional Chinese Medicine, Jinan, China; ^6^ Department of Oncology, Weifang Traditional Chinese Hospital, Weifang, China

**Keywords:** T cell exhaustion, chronic inflammation, tumor immune escape, immunotherapy, tumor microenvironment

## Abstract

The development and response to treatment of tumor are modulated by inflammation, and chronic inflammation promotes tumor progression and therapy resistance. This article summarizes the dynamic evolution of inflammation from acute to chronic in the process of tumor development, and its effect on T cells from activation to the promotion of exhaustion. We review the mechanisms by which inflammatory cells and inflammatory cytokines regulate T cell exhaustion and methods for targeting chronic inflammation to improve the efficacy of immunotherapy. It is great significance to refer to the specific state of inflammation and T cells at different stages of tumor development for accurate clinical decision-making of immunotherapy and improving the efficiency of tumor immunotherapy.

## 1 Background

There seems to be a role for chronic inflammation in modulating all phases of malignant disease, including morbidity and mortality (1). There is substantial evidence that chronic inflammation caused by persistent infections, autoimmune reactions, or exposure to toxic chemicals increases the risk of tumor (2). However, not all chronic inflammation leads to tumors, the tissue where chronic inflammation occurs also affects cancer development. Inflammation of the gut or liver can greatly increase the risk of tumor, while inflammation of the joints or muscles rarely affects the development of tumor (3).

Tumor-associated inflammation underwent a transition from acute inflammation to chronic inflammation. Acute inflammation is a protective response elicited by injury and infection, being positive for immune activation. While, chronic inflammation supports immunosuppression rather than immune activation, thereby resulting in the growth and progression of tumors (4). Acute inflammation makes a difference in tissue regeneration and plays an important immunostimulatory function (5). However, due to the persistence of damage, a repair can not be completed in time, and inflammation is transformed from acute inflammation to chronic inflammation. Chronic inflammation can lead to genetic mutations and epigenetic changes in normal tissues that drive malignant transformation (6).

Largely due to the exhaustion of T cells, chronic inflammation inhibits immunity, leading to tumor progression. T cell exhaustion is one of the mechanisms that tumor cells are able to escape from immune control (7).

As monoclonal antibodies are increasingly used in tumor medicine, more options are available across a wide range of oncological indications. Some patients, nevertheless, may not benefit from an inhibitor of immune checkpoint receptors. Even patients who benefit do not last. It appears that tumor-induced inflammation is an important driver of malignant progression in many solid malignancies. A chronic inflammatory response is a significant contributing factor to a majority of solid and hematopoietic tumors (3). Immune-related adverse events (irAEs) are also significant factors that limit the use of immune checkpoint blockers besides drug resistance in patients who are suitable for immunotherapy (8). T-cell exhaustion caused by chronic inflammation is a negative feedback regulator in response to infection and tissue damage. Immune-related adverse reactions occur partly because immunotherapy disrupts this regulatory mechanism (9). Immune checkpoint blockers partially reversed T cell exhaustion, breaking this negative feedback mechanism, increasing the immunotherapeutic efficiency. It also unties the reins of immune cells and increases the damage of immune cells to normal tissues, resulting in serious immune-related events. Therefore, it is greatly significant to deeply learn the complex interaction between the chronic inflammation microenvironment and immunity to improve the effect of immunotherapy and reduce related adverse events.

In addition to immunotherapy, traditional radiotherapy and chemotherapy remain clinically mainstream. However, radio-chemotherapy induced dying cells are of great concern, a variety of cell death patterns may occur, as well as the release of complex factors, and promote the occurrence of chronic inflammation in the tumor microenvironment, thus orchestrating repopulation cascades of tumor (10). Dead cells enhance the survival of residual viable tumor cells, boost proliferation, and hasten tumor cell metastasis (11). Therefore, further study of tumor-related chronic inflammation and finding appropriate intervention targets is not only necessary to improve the effect of immunotherapy, but also necessary to improve the long-term therapeutic effect of radiotherapy and chemotherapy.

## 2 T cell exhaustion

For tumor cells, the immune cells that play the main effect are CD8+ T cells, but due to the chronic inflammatory continuous stimulation, the killing function of T cells will gradually decline, presenting a state of exhaustion. T cell exhaustion is the body’s self-protective mechanism to prevent excessive immunity from damaging normal tissues in the tumor microenvironment. T cell exhaustion is a compromise of the autoimmune system for the repair of long-standing viral, bacterial infections, or chronic tissue damage machine-processed. In these settings, perhaps (partial) T cell exhaustion strikes the balance between maintaining limited infection control capacity and moderating immuno-pathology. For a long time, exhaustion is thought to represent a series of cellular dysfunctions. Exhausted cells do make a significant contribution to infection control, although they can not clear it all (12).

### 2.1 Characteristics of the T cell exhaustion

The loss of function in exhausted CD8+ T cells occurs hierarchically, with some properties being lost before others (13). Usually, the first thing that gets lost in exhausted T cells is their ability to produce IL-2 and to proliferate. Other properties are lost during the intermediate stages of cell exhaustion, including the ability to produce tumor necrosis factor. When cells become severely exhausted, they can no longer produce large quantities of interferon-gamma (IFN-γ) or beta-chemokines or to degranulate. When T cells are completely exhausted, they become exhausted T cells with total disappearance of their effector functions (14). Exhaustion is often consistent with the expression of inhibitory surface receptors, including PD1, CD160, 2B4, LAG-3, and CTLA-4 (7). Poor effector function and persistent expression of inhibitory receptors are key features of T cell exhaustion (15).

### 2.2 Inflammatory regulation of T cell exhaustion

In the inflammatory microenvironment, T cell failure is regulated by inflammatory factors (IL-10/IL-35/IL-7/IL-21/TGF-β, etc.) (16) inflammatory cells (Treg, MDSCS, macrophages, neutrophils, mesenchymal cells, etc.), and related nutrients (glucose, amino acids, fatty acids, etc.) (12). The specific mechanism of action will be discussed in the following article.

## 3 Sources and effects of inflammation in tumor development

In tissues, inflammation occurs earlier than in tumors. Local inflammation is largely caused by infection or the stimulation of environmental factors, and systemic inflammation is mostly related to obesity and metabolic diseases. Both local and systemic inflammation interact through the circulatory system. The tumor and related radiotherapy and chemotherapy can promote both local inflammation and systemic inflammation. During tumor progression, inflammation undergoes a transition from acute to chronic, and its effect on T cells also switches from activation to promotion of exhaustion. It is very essential to understand how inflammation transforms in different stages of tumor growth to improve the effectiveness of immunotherapy.

### 3.1 Inflammation and precancerous lesions

#### 3.1.1 Local inflammation

Pathogen microbial infection and local stimuli are the main causes of chronic local inflammation, such as Helicobacter pylori infection, Hepatitis virus infection pancreatitis, colitis, esophagitis, cholangitis, etc. (17). Environmental and chemical human carcinogens induce pro-tumor inflammation, including UV, aflatoxins, nitrosamines, and tobacco (18, 19). Local chronic inflammation activates the NF-κB pathway, which suppresses apoptosis with malignant potential, leading to a malignant transformation in the tissue (20). The activation of NF-κB plays a vital role in the control of the communication between tumor cells and inflammatory cells (21). The microenvironment of a tumor and normal tissues are always filled with activation signals. NF-κB pathway activation occurs when p53 is dysfunctional, issuing in increased expression of inflammatory genes (22). NF-κB activity can be induced by many factors, such as TNFα, IL-1β, LPS, ionizing radiation, ROS, etc. (23). The ROS produced by neutrophils and macrophages can not only activate the NF-κB pathway, but also cause DNA disruption, induce gene mutations, and increase susceptibility to tumors (24). In addition, in the chronic inflammatory microenvironment, the formation of tumor progression is conducive to the immune suppression microenvironment, this inhibitory microenvironment will not only lead to immune escape but also a screening of tissues, only to adapt to this inhibitory environment of malignant cells that can survive, this selection process becomes immune editing (25). In conclusion, chronic inflammation plays an important role in the malignant change of cells, and later immune escape.

#### 3.1.2 Systemic inflammation and precancerous lesions

Systemic inflammation can evolve from local inflammation or result from systemic metabolic diseases. The local immune response consists of cytokines derived from a tumor and inflammatory proteins derived from the host, as well as infiltrating immune cells, acting in the local tumor micro-environment (26). On the other hand, systemic inflammation is also associated with small molecules in local inflammation. The difference is that these mediators flow in the systemic circulation and lead to paraneoplastic syndromes in cancer patients. It is evident that mediators in the systematic inflammatory microenvironment and the local microenvironment communicate extensively (26).

As a result of adipose expansion and chronic obesity, an inflammatory program is activated, permanently skewing the immune system in favor of inflammation (27). There is ample evidence that obesity is closely associated with colon tumors, oesophageal tumors (adenocarcinoma), renal tumor (renal cell carcinoma), breast tumor (postmenopausal), and endometrial tumor (28).

Adipose tissue necrosis causes macrophage infiltration, while the metabolism of adipose tissue itself, produces chemokines and resident factors, causing the indwelling of macrophages. Invasive macrophages produce inflammatory factors to further shape the inflammatory microenvironment. In addition to TAMs, MDSCs and DCs are also recruited by adipose tissue, both of which can promote chronic inflammation in the context of obesity (29, 30). In adipose tissue, the number or proportion of Treg cells decreases will further promote the development of chronic inflammation (31). The adipose tissue is directly resulted from the nutrient excess, which also leads to reactive oxygen species (ROS) production sourced from mitochondrial (32). ROS stimulates chronic inflammation by activating the upstream kinases I-κB and JNK to provoke proinflammatory transcription factors, such as AP-1 and NF-κB (33). Inflammation in adipose tissue may also be influenced by commensal flora metabolism, through bacterial products (34). The inflammation caused by adipose tissue is chronic, and metabolic. It does not disappear unless the adipose tissue disappears.

According to the current study, the gut microbiota and its metabolites can trigger inflammation in obese and diabetic individuals. The chronic inflammatory response further increases insulin resistance, and the two promote each other, forming a bad cycle (35).

### 3.2 Inflammation and tumor

**Figure 1:**
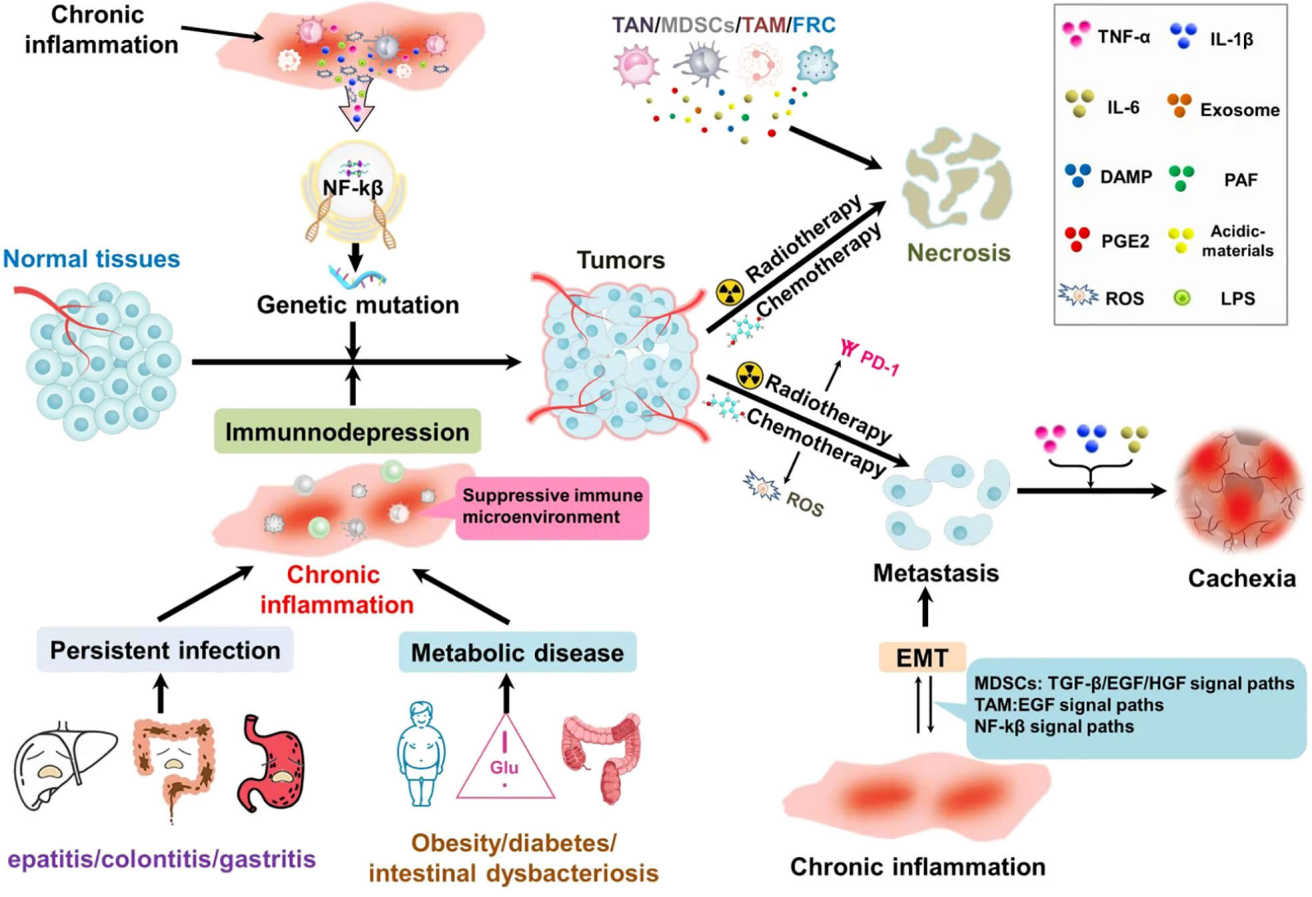
Chronic inflammation of different sources accompanies the whole process of tumor development: in precancerous lesions, chronic local irritation, and chronic inflammation caused by systemic metabolic diseases not only activate the NF-κB pathway, but also induce gene mutations that lead to tissue malignant transformation. At the same time, it can inhibit the immune monitoring and make the tumor cells escape. Tumor treatment could result in the necrosis of tumor cells. Necrosis of the tumor cells chemotactic inflammatory cell aggregation, suppression of immunity; At the same time, a large number of cytokines are released to generate factor storms leading to the aggregation and recurrence of tumor cells. Chronic inflammation and epithelial-mesenchymal transformation promote each other and form a vicious cycle, which is conducive to distant metastasis of tumors. Inflammatory factors are closely related to the production of tumor cachexia.

Chronic inflammation plays an important role in the occurrence, development, distant metastasis, and recurrence of most tumors (**Figure 1**). That is, the occurrence of certain tumors and inflammation are not closely related, but the treatment of inflammation to intervene, can still achieve positive efficacy (36, 37).

#### 3.2.1 The dynamic evolution of tumor, inflammation and immunity

Acute inflammation is caused by the recognition of pathogen-associated molecular patterns (PAMPs), or damage-associated molecular patterns (DAMPs), which activate pro-inflammatory cytokines and chemokines, thereby enhancing immune responses (38). Granulocytes constitute the majority of infiltrating inflammatory cells in acute inflammation (39). Acute inflammatory responses typically stimulate dendritic cell (DC) maturation and antigen presentation, favoring the activation of CD8+ T cells. During the tumor, acute inflammation mostly occurs in the early stage of the tumor (40), which is caused by tumor-specific antigens or tumor-associated antigens (41). Acute inflammation can also be led by tumor growth and invasion to destroy normal tissue (42), tumor-specific chemotherapy and low-dose, radiation (43).

The simultaneous occurrence of tissue destruction and repair is a hallmark of chronic inflammation. The main infiltrating immune cells at sites of chronic inflammation are macrophages and lymphocytes (44). Chronic inflammation leads to the presence of high amount of immunosuppressive cells (TAM_2_,Treg cells, MDSCs etc.) and cytokines in the tumor microenvironment (45), which induces T cell exhaustion and produces an immunosuppressive microenvironment. Before the occurrence of tumors, chronic inflammation already exists and produces immunosuppression, which is a risk factor for tumorigenesis. In the process of the tumor, most solid tumors develop chronic inflammation that promotes tumor progression (45). Tumor cells and stromal cells release chemokines, recruit macrophages and neutrophils (46), tumor growth and invasion can damage normal tissues and release damage-associated molecular patterns that activate granulocytes (42). Tumor-specific metabolic patterns result in an acidic and hypoxia TME, leading to neovascularization and recruitment of macrophages (47).

#### 3.2.2 Inflammation and tumor necrosis

Chemoradiation-induced death of tumor cells releases complex factors, thereby coordinating the cascade of tumor regeneration. Dead cells enhance the survival of residual live tumor cells and promote metastasis. In addition, dead cells can also contribute to chemoradiation-induced side effects (11, 48). Cells dying from chemoradiotherapy also secrete platelet activation factor (PAF)-like phospholipids, which further exacerbate immunosuppression by binding to the PAF receptor (49, 50). Tumor-related therapy can also lead to drug resistance in patients (51).

#### 3.2.3 Inflammation and chemotherapy

Chemotherapy can trigger inflammation through ROS and damage-related proteins (DAMP) (52). Chemotherapy or immunotherapy damages the normal tissue, thereby inducing the migration of macrophages, and cytotoxic lymphocytes to damaged and non-damaged sites, causing chronic inflammation of the tissue (53). Tumor treatment will also promote the production of ROS (54). Reactive oxygen species result in mitochondrial DNA (mtDNA) and nuclear DNA damage. DAMP from the mitochondria enters the cellular cytoplasm, activates intercellular spatial receptors, and further triggers the recruitment of immune effector cells (55). Computer modeling study shows that increased tumor cell death from chemoradiotherapy leads to short-term tumor shrinkage, but ultimately can accelerate tumor growth and metastasis in the long-term (56).

#### 3.2.4 Inflammation and radiation therapy

Antitumor immunity can be stimulated by low-dose radiation therapy (57). Radiotherapy can contribute to the expression of proinflammatory chemicals, cytokines, and NK cells, stimulate the formation of inflammation and be conducive to activating the immune response (58). High-dose radiotherapy acts directly on sphingal membrane-bound sphingolipid, decomposes sphingolipid, and generates ceramide by sphingingholipase. This process results in the accumulation of ceramides in damaged tissues. Angiogenesis is suppressed by ceramides, which damage endothelial cells (59). Immune cells are less likely to be attracted to damaged/inflammatory regions through blood flow areas when angiogenic inhibition is present. It is a failure to recruit immune cells (macrophages, T cells, and endocytic) resulting in PD-L1 accumulation and immunosuppression.

#### 3.2.5 Inflammation and metastasis

EMT is important for tumor metastasis. The EMT program not only enables tumor cells to exhibit enhanced invasiveness, stem cell-like characteristics, and resistance to apoptosis, but also stimulates tumor cells to produce pro-inflammatory factors. At the same time, inflammation further induces EMT. Therefore, in an alliance of metastatic growth, these phenomena may support each other (60). In the tumor microenvironment, inflammatory cells can effectively promote EMT. Macrophages induce EMT by the production of EGF (61). Bone marrow-derived suppressive cells infiltrate the primary tumor and induce EMT in tumor cells through the TGF-β, EGF, and HGF signaling pathways, leading to metastasis and the spread of tumor cells (62). In addition to inflammatory cells, many inflammatory factors also play important roles in EMT, such as TGF-β、IL-6、CCL-20/CCL-8 et al. (63–65). In conclusion, chronic inflammation not only suppresses the anti-tumor immunity but also promotes distant tumor metastasis by promoting EMT.

#### 3.2.6 Inflammation and cachexia

Tumor cachexia, also known as wasting syndrome, is closely associated with chronic inflammation. Cachexia is characterized by severe atrophy of skeletal muscle and gradual weight loss due to partial adipose tissue. Epidemiological data suggest that circulating CRP levels are associated with cachexia levels and mortality risk in patients. Inflammatory cytokines such as IL-6, IL-1β, and TNF-α are boosted in the TME in patients with systemic or cachexia (66). Pro-inflammatory factors have direct causal relationships with cachexia. TNF-α, tumor proteolytic inducible factors and lipid mobilization factors are directly relevant to the breakdown of muscle and adipose tissue (67). Other pro-inflammatory cytokines, including IL-1 and IL-6 induced a strong reduction in transporters involved in bile formation and bile acid secretion, further aggravating the development of cachexia (68).

## 4 Chronic inflammation and T cell exhaustion

**Figure 2:**
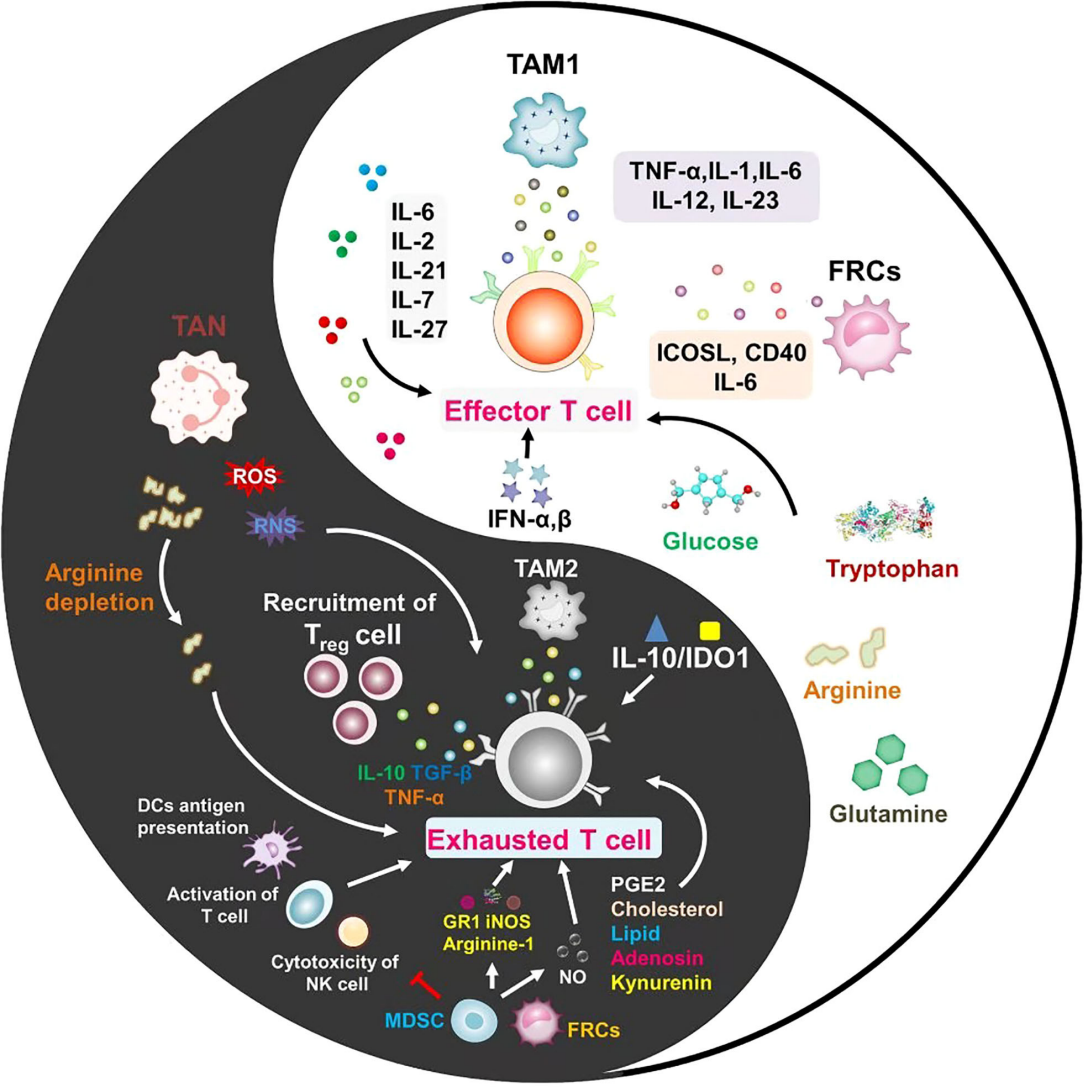
Every coin has two sides, and inflammation is no exception. In the tumor microenvironment, the role of inflammatory cells and inflammatory factors on T cells is not static. The different mechanisms between inflammatory factors for promoting T cell activation or exhaustion are important issues to be considered in future studies.

Different aspects of inflammation seem to play a role in all stages of malignant disease. Although acute inflammation can stimulate immunity, tumor-associated inflammation suppresses immunity and promotes tumor progression (69). The suppression of chronic inflammation on immunity is mainly reflected in the induction of T cell exhaustion, so this section discusses in detail how chronic inflammation regulates T cell exhaustion (**Figure 2**). It is concluded that the signals regulating T cell exhaustion are mainly divided into the following three categories. Sustained antigenic stimulation (signal 1) from viruses or tumor cells is a central factor in T cell exhaustion (70). Proinflammatory cytokines and inhibitory cytokines (signal 2) produced by virus-infected cells, immunoregulatory cells, and tumors are important players in regulating T cell exhaustion in chronic inflammation. Inhibitory receptors on the surface of T cells (signal 3) provide a negative costimulatory signal, which blocks the activation of T cell effectors and renders T cells unable to mount a robust immune response (71).

### 4.1 Mechanism of T cell exhaustion

It has been conducted for the research on immunosuppressive receptors such as PD-1, TIM-3, LAG3, CTLA-4 and BTLA (72). However, there are still few studies on the mechanisms regulating receptor and ligand expression in tumor-associated chronic inflammation. The expression of inhibitory receptors and their ligands plays a critical role in T cell exhaustion (73).

It is one of the hallmarks of T cell exhaustion that the expression of inhibitory receptors on the surface of T cells. The nuclear factor of activated T cells (NFAT) controls CD8+ T cell exhaustion by binding to the promoter of exhaustion-related genes like Pdcd1 (PD-1) and Havcr2 (TIM3) (74). STAT3/STAT4 can induce the expression of PDCD1 by acting on the transcription factor NFAT (75). This may partly explain the specific mechanism how chronic inflammation induces T cell exhaustion (76). Nuclear receptor 4A (Nr4a) and Thymocyte selection-associated high mobility group box protein (Tox) transcription factors promote T cell exhaustion by acting on NFAT. Tox2 transcription factor and Nr4a transcription factor may be promising targets for future immunotherapy (77). T-bet directly inhibits the transcription of PD-1-encoding genes, resulting in decreased expression of other inhibitory receptors. Sustained antigenic stimulation leads to T-bet downregulation, leading to more severe exhaustion of CD8+ T cells (78). The progression of T cell exhaustion is associated with additional repressor genes and transcriptional pathways, such as Forkhead box transcription factor O1 (Foxo1) (79), B lymphocyte-induced maturation protein 1 (Blimp-1) (80), Basic leucine zipper transcription factor ATF-like -interferon-regulatory factor (BATF-IRF) interaction (79).

Inhibitory ligands on the surface of immune cells and tumor cells are also important factors affecting T cell exhaustion. Hyperactivated ALK signaling pathway promotes PD-L1 expression *via* STAT3 which is activated by NPM-ALK gene fusion (80). Other pathways that are associated with chronic inflammation such as the NF-kB pathway, PTEN/PI3K pathway and MAPK pathway are all involved in the regulation of PD-L1 expression (81–84).

### 4.2 Chronic inflammation regulates T cell exhaustion

In conclusion, soluble microkinis have an important role to play in regulating immunity (**Table 1**), in inflammatory microenvironment and even in a sense, the composition of different types of cytokines determines the quality of T cell pathogen-specific response. Inflammation-related factors can not only induce the expression of inhibitory receptors on the T cell surface, but also activate oncogenic pathways and alter the expression of inhibitory ligands (**Table 1**). Notch signaling promotes T cell exhaustion by affecting NFAT transcription factors (103). Notch signaling is involved in both malignancies and chronic inflammatory diseases (104, 105). IL-6 can promote T cell exhaustion through IL-6/STAT3/PD-1 transcription regulation (106). Other pro-inflammatory cytokines in TME promote the expression of PD-1 on the surface of T cells, leading to the exhaustion of T cells such as Il-10,TGF-β (106–109). Inflammatory factors (IFN-a, IFN-b, TNF-α, TGF-β) can also induce PD-L1 expression and attenuate immune cell activation (110).

**Table 1:** Factors in chronic inflammation.

Factors	Effects on immune cell	Refs
TNFα	Active the NF-κB pathway, Promote expression of PD-L1	(85–87)
TGFβ	Acts as an important mechanism of immune evasion Induce the expression of pd-l1	(88–90)
IFNα/β	Activate of innate immune cells Induce the expression of negative regulators, Mediate T cell death through Fas/Fasl	(91, 92)
IL-10	Induce t cell exhaustion by BLIMP1	(93, 94)
IL-6	Improve the action of CD4+ T cells Inhibit the differentiation of Treg cells Promote TH17 differentiation	(95, 96)
IL-2	Maintain memory CD8+ T cells	(97)
IL-21	Induce BATF expression	(98, 99)
IL-7	Regulate the mechanisms of memory T cell survival	(100, 101)
adenosine	Inhibit T cell function through A2AR	(102)

Inflammation-related cells can promote T cell exhaustion, possibly through cell-surface immunosuppressive ligands or by self-secreting inhibitory inflammatory factors (**Table 2**). Immunosuppressive ligands on the surface of tumor cells and macrophages (127), bind to receptors on the T cell surface, inhibiting the killing function of T cells and promoting the exhaustion of T cells (128). TAMs and MDSCs were associated with the expression of PD-1 on the T cell surface (129, 130). Treg cell-derived IL-10 and IL-35 promote BLIMP1-dependent depletion of CD8+ TILs, thereby limiting effective antitumor immunity (94).

**Table 2:** Immune cells in chronic inflammation.

Immune cells	Outcome	Refs
TAMs	Release immunosuppressive molecules Express the ligands of PD-1, PD-L1, PD-L2	(111–116)
Treg cells	Release immunosuppressive molecules Induce DCs to express immunosuppressive molecules Express cytotoxic T lymphocyte antigen 4 (CTLA4) Produce adenosine, Inhibit tumor-associated antigen presentation, Inhibit cytolytic granule release	(114, 117, 118)
MDSCs	Disrupt dendritic cells (DCs) antigen presentation Inhibiting cytotoxicity of NK cells Express high levels of arginase I Induce nitric oxide synthase (iNOS), and GR1	(119, 120)
TANs	Produce ROS,RNS and angiogenic factors Increases inhibitory receptor expression on T cells Recruitment of Treg Release arginine-1/neutrophil external traps (NETs)	(121–124)
Mesenchymal cells	Induce NO Express co-stimulatory molecules	(125, 126)

### 4.3 The mechanism of inhibitory receptor/ligand

When the inhibitory receptor on the surface of T cells binds to the corresponding ligand, it can block the binding of other stimulatory ligands and inhibit the activation of T cells (131). Inhibitory receptors can induce a decrease or disappearance of the expression of intracellular receptors that positively regulate immunity or costimulatory factors (132). In addition, inhibitory receptors induce the expression of certain inhibitory genes that promote T cell exhaustion (133).

## 5 Methods to reverse T cell exhaustion by targeting inflammatory microenvironment

As tumor treatment enters the immune era, the application of immune checkpoint blockers has become more and more extensive. T-cell exhaustion is a negative feedback mechanism in the body, and immune checkpoint blockers directly disrupt this negative feedback regulatory state, sometimes resulting in immune cells destroying normal tissues in the body. This phenomenon is known as immune-related adverse reactions, which can sometimes be more deadly than tumors. Therefore, it is greatly important for the safe application of immunotherapy to keep the balance between the state of T cells and the body’s protective mechanism.

Moreover, immune checkpoint blockers can partially reverse T cell exhaustion, but sometimes the treatment is not durable. One of the reasons for this is the continuous stimulation of the chronic inflammatory environment in the tumor (131).

For the T cell exhaustion induced by chronic inflammation, researchers have developed many treatments that are combined with immune checkpoint blockers to improve treatment responsiveness and persistence of efficacy. The first is the simultaneous use of multiple immune checkpoint blockers (134). Since T cell failure is not only manifested by elevated PD-1 expression, other immune checkpoints also make a difference. The simultaneous application of multiple immune checkpoints blockers can not only directly reverse the T cell exhaustion, but also increase the glucose uptake and utilization ability of T cells, thus conducive to the activation of T cells (135).

While applying immunotherapy, the combination with traditional radiotherapy can also increase the effect of immunotherapy. Radiation therapy can increase the infiltration of T cells while causing acute inflammation can enhance cellular immunity (136). Certain chemotherapeutic drugs or radiation can induce immunogenic death of tumor cells, followed by the release of tumor antigens which are recognized by antigen-presenting cells and facilitate the activation of T cells (137).

Both local and systemic chronic inflammation increases the incidence of malignancies and is closely associated with a poor prognosis (27). Anti-inflammatory treatment (**Table 3**) and the treatment for metabolic diseases are beneficial to prevent tumors; the combined application of immune checkpoint blockers is expected to enhance therapeutic efficiency of immune checkpoint blockers and delay tumor progression (143).

**Table 3:** Anti-inflammatory treatment are beneficial to prevent tumors.

Anti-inflammatory treatment	Outcomes	Refs
Aspirin	Reduce risk of colorectal tumor, and possibly of a few other digestive tract tumors	(138)
Indomethacin	Promote SYVN1-mediated ubiquitination of ITGAV, and potentiating cytotoxic CD8 T cell responses	(139)
Metformin	Induce activation of the JAK1/2/3/STAT5 and AKT/mTOR pathways in a p38 MAPK-dependent manner	(140)
Statins	Activate antigen-presenting cells and tumor-specific CD8+ T cells	(141)
The dietary patterns	Lower the risk of developing several tumors	(142)

In addition to systemic metabolism, the lack of amino acids, glucose, cholesterol, and the accumulation of lactate and lipid can also affect T cell exhaustion, and targeting these metabolic targets for treatment will also strongly promote T cell activation (144). Immune checkpoints inhibit T cell function by inhibiting glycolysis in immune cells. So immune checkpoint blockers can restore glucose uptake by immune cells (145). Blocking the tumor metabolism of glutamine not only brings about reduced hypoxia, acidosis, and nutrient consumption in the tumor microenvironment but also promotes T cell activation and extends the life span of T cells (146). Targeted arginase (ARG) restores arginine levels, leading to tumor regression and improved T cell function (147). The combination of the ARG1 (arginase 1) targeted vaccine with anti-PD-1 also leads to increased T cell infiltration and promotes T cell activation (148). Adenosine-producing enzyme CD73 is inhibited by mAb19 and adenosine-mediated T cell exhaustion is suppressed *in vitro* by mAb19 (149).

Strategies that target fatty acid synthases or fatty acid oxidation to improve lipid abundance and thereby improve immune efficacy are beneficial in mouse tumor models (150). The accumulation of lactic acid can inhibit immunity, and one way to target lactic acid is to inhibit lactic acid transporters to reduce lactate acid accumulation (151).

Cytokines in the inflammatory microenvironment have different directions on T cell exhaustion. Increasing factors that antagonize T cell exhaustion or blocking factors that promote T cell exhaustion, combined with immune checkpoint blockers, has been actively explored in anti-tumor therapy. During chronic viral infection, increasing the IL-2 or IL-7 content in the inflammatory microenvironment can improve the virus-specific CD8+ T cell response and accelerate viral clearance (152, 153). Blocking the interleukin-10 receptor increases the effect of CD8+ T cells and accelerates the rate of virus clearance (154). The blockade of TGF-β, TNF-α, also showed meaningful results in reversing T cell exhaustion (155, 156).

In addition to targeting their secreted inflammatory factors, direct intervention in the polarization and recruitment of inflammatory cells is also a promising therapeutic modality. Reduction of Treg cells or block of Treg cell differentiation may allow CD8+ T cells to increase their immune surveillance of tumor cells (157). Targeting MDSCs with monoclonal antibodies restored the function of TILs (119). Colony-stimulating factor 1 (CSF-1) as a signal for macrophage recruitment and aggregation, using a small molecule PLX3397 that effectively inhibits the activity of the CSF-1R tyrosine kinase, can effectively reduce macrophage recruitment and improve tumor recurrence (158). In addition to blocking recruitment, M2 macrophages can be reeducated. It has been shown that blocking NF-κB activation in TAMs can convert TAMs from a tumor-promoting M2 phenotype to an m1-similar cytotoxic phenotype, thereby alleviating immunosuppression and enhancing the tumor control effect (159). Neutrophils are able to promote angiogenesis and promote distant tumor metastasis. Targeted granulineolytic colony-stimulating factors (G-CSF) can inhibit the recruitment of neutrophils and enhance the efficacy of antiangiogenic agents (160). The combination of therapy targeting the inflammatory microenvironment with immune checkpoint blockers is expected to further improve the efficiency of immunotherapy in clinical application.

## 6 Discussion

Cancer development and its response to therapy are modulated by chronic inflammation, and chronic inflammation promotes tumor progression and therapy resistance. We discuss in detail how chronic inflammation induces T cell exhaustion in tumors and the prospects of targeting chronic inflammation in combination with immune checkpoint blockers for tumor therapy.

In addition, this article summarizes the dynamic evolution of inflammation and the results of different effects on the body’s immunity during tumor development. Discuss the positive role of inflammation and T cell exhaustion in maintaining health from a new perspective. The research in this paper has important reference significance for making the accurate clinical decision in different stages of tumor development, by combining with the specific state of inflammation and T cells.

In the treatment of reversing T cell exhaustion, in addition to directly using immune checkpoint blockers to act on CD8+ T cells, targeting related helper cells can also effectively promote T cell activation. At present, there is further research on the exhaustion of CD4+ T cells, which is beneficial to stimulating stronger anti-tumor immunity (161). Fibroblasts retain an effective inflammatory environment in chronic inflammation, resulting in immunosuppression in malignant tumors (162). Future studies could further focus on the link between associated helper cells and T cell exhaustion, providing more targets for reversing T cell exhaustion.

Inflammation factors in the tumor microenvironment play an dual-directional and dynamic role in the regulation of T cell exhaustion (163). IL-2 has a dual role in regulating tumor-associated immunity (164). IL-2 can promote the activation and proliferation of CD8+ T cells in the early stage of tumor, but it can switch its function to induce the exhaustion of CD8+ T cells in the late stage of tumor. This change may be related to the increase of IL-2 content (165). Oncostatin (OSM), as a member of the Il-6 family, also has two-sided effects on immunity. In the early stages of tumors, OSM can activate immunity and inhibit tumor progression (166). However, in the late stage of the tumor, it will inhibit the immune system and promote the progression of the tumor (167). The mechanism of this effect may be related to the activation of JAK-STAT signaling pathway (168). It may also be due to the accumulation of the concentration (169), which has transformed its effect. The tumor microenvironment is a small ecosystem (170), and the interaction of tumor cells with immune cells and cytokines cannot be viewed in isolation (171).

Bioinformatics and single-cell sequencing technologies are playing an increasingly important role in tumor research. Under the background of the era of precision therapy, it is very necessary to apply relevant analysis techniques to perform accurate subgroup analysis and marker analysis for T cell exhaustion. Therefore, for the degree of T cell exhaustion and the evaluation of the effect of immune checkpoint blockers therapy, it is necessary to further study for related markers to clarify the indication of the application of immune checkpoint and the timing of stopping the immune checkpoint inhibitors, so as to improve the anti-tumor efficacy and reduce the damage of excessive immunity to the body (146). With the application of single-cell sequencing, recent studies have defined the heterogeneity of T lymphocyte populations at the genetic level (150). This approach may be used to monitor the effects of different immunotherapies on specific subsets of TIL and specific diseases. This paves the way for the use of blockers at specific immune checkpoints in the future.

The gut microbiota directly or indirectly affects the differentiation and function of immune cells, and the gut microbiota is a promising target for the treatment of inflammation-related cancers. Little is known about the mechanisms by which gut microbiota regulates T cell exhaustion. It is believed that the intervention of intestinal flora can further improve the effect of immunotherapy.

There are many studies on the mechanism of inhibitory receptors on T cells, but little is known about how the expression of inhibitory receptors is regulated in the tumor microenvironment. This also indirectly leads to less research on the specific regulatory mechanism of inflammatory mediators and inflammatory cells intervening in T cell exhaustion, only focusing on the correlation between inflammatory factors or cells and the expression of inhibitory receptors. There are some studies on the regulation of T-bet and NFAT on the expression of immunosuppressive receptors. While relative to the complex inhibitory receptors, the current research is still insufficient. Moreover, the current mechanism research is still far from the clinical application of targeted inhibitory receptor combined immunotherapy. Therefore, future research can focus more on the mechanism of the tumor microenvironment regulating the expression of immunosuppressive receptors on the basis of modern technologies such as bioinformatics and single-cell sequencing, combined with existing experimental techniques.

## Author contributions

LF and CS conceived and designed this review. LF and KL wrote the paper. CL, XW, WM, WX, and JW provided direction and guidance throughout the preparation of this manuscript. All authors contributed to the article and approved the submitted version.

## Funding

This work was supported by the National Natural Science Foundation of China (81973677; 82174222) and Shandong Province Natural Science Foundation (ZR2021LZY015, ZR202103030292).

## Conflict of interest

The authors declare that the research was conducted in the absence of any commercial or financial relationships that could be construed as a potential conflict of interest.

The reviewer QW declared a shared affiliation with the author WM at the time of review.

## Publisher’s note

All claims expressed in this article are solely those of the authors and do not necessarily represent those of their affiliated organizations, or those of the publisher, the editors and the reviewers. Any product that may be evaluated in this article, or claim that may be made by its manufacturer, is not guaranteed or endorsed by the publisher.

## Abbreviations

TILs, Tumor-infiltrating T lymphocytes; TAM, Tumor-associated macrophages; TAN, Tumor-associated neutrophils; MDSCs, Myeloid-derived suppressor cells; DC, Dendritic cells; Treg, T regulatory cell; FRC, Fibroblast reticulate cells; TRCs, T region reticulate cells; CTLA4,T lymphocyte antigen 4; PD-L1, Programmed death ligand-1; PD-1, Programmed death-1; DAMPs, Damage-related proteins; PAMPs, Pathogen-associated molecular patterns; TGFβ, Transforming growth factor-beta; TNF-α, Tumor necrosis factor-alpha; PGE2, Prostaglandin E2; ROS, Reactive oxygen species; RNS, Reactive nitrogen; LPS, Lipopolysaccharides; EMT, Epithelial-to-mesenchymal transition; EGF, Epidermal growth factor; NETs, Neutrophil external traps; IDO1, Indindamine-2,3-dioxygenase; iNOS, Inducible Nitric oxide synthase; TLR5, Toll-like receptor 5; PAF, Platelet activation factor; TME, Tumor microenvironment; TSP1, thrombospondin-1; ARG, Arginase; CSF-1, Colony-stimulating factor 1; GPCRs, G protein-coupled receptors; TDAG8, T cell death-associated gene 8; OGR1,Ovarian tumor G-protein-coupled receptor 1; mtDNA, Mitochondrial DNA; TCA cycle, Tricarboxylic acid cycle; T2D, Type 2 diabetes; TOX, thymocyte selection-associated high mobility group box protein; Nr4a, Nuclear receptor 4A; Foxo1, Forkhead box transcription factor O1; Blimp-1, B lymphocyte-induced maturation protein 1; BATF, The basic leucine zipper transcription factor activating transcription factor-like; IRF, interferon-regulatory factor; OSM, Oncostatin.
